# Toward elimination of mother-to-child transmission of HIV in Malawi: Findings from the Malawi Population-based HIV Impact Assessment (2015–2016)

**DOI:** 10.1371/journal.pone.0273639

**Published:** 2022-09-01

**Authors:** Evelyn Kim, Sasi Jonnalagadda, Juliana Cuervo-Rojas, Andreas Jahn, Danielle Payne, Christine West, Francis Ogollah, Alice Maida, Dumbani Kayira, Rose Nyirenda, Trudy Dobbs, Hetal Patel, Elizabeth Radin, Andrew Voetsch, Andrew Auld

**Affiliations:** 1 Division of Global HIV & Tuberculosis, U.S. Centers for Disease Control and Prevention, Lilongwe, Malawi; 2 Division of Global HIV & Tuberculosis, U.S. Centers for Disease Control and Prevention, Atlanta, GA, United States of America; 3 ICAP at Columbia University, New York, NY, United States of America; 4 Faculty of Medicine, Pontificia Universidad Javeriana, Bogotá, Colombia; 5 Division of HIV and AIDS, Ministry of Health, Government of Malawi, Lilongwe, Malawi; PhD, PLOS, UNITED KINGDOM

## Abstract

**Background:**

Malawi spearheaded the development and implementation of Option B+ for prevention of mother-to-child transmission of HIV (PMTCT), providing life-long ART for all HIV-positive pregnant and breastfeeding women. We used data from the 2015–2016 Malawi Population-based HIV Impact Assessment (MPHIA) to estimate progress toward 90-90-90 targets (90% of those with HIV know their HIV-positive status; of these, 90% are receiving ART; and of these, 90% have viral load suppression [VLS]) for HIV-positive women reporting a live birth in the previous 3 years.

**Methods:**

MPHIA was a nationally representative household survey; consenting eligible women aged 15–64 years were interviewed on pregnancies and outcomes, including HIV status during their most recent pregnancy, PMTCT uptake, and early infant diagnosis (EID) testing. Descriptive analyses were weighted to account for the complex survey design. Viral load (VL) results were categorized by VLS (<1,000 copies/mL) and undetectable VL (target not detected/below the limit of detection).

**Results:**

Of the 3,153 women included in our analysis, 371 (10.1%, 95% confidence interval [CI]: 8.8%–11.3%) tested HIV positive in the survey. Most HIV-positive women (84.2%, 95% CI: 79.9%–88.6%) reported knowing their HIV-positive status; of these, 94.9% (95% CI: 91.7%–98.2%) were receiving ART; and of these, 91.2% (95% CI: 87.4%–95.0%) had VLS. Among the 371 HIV-positive women, 76.0% (95% CI: 70.4%–81.7%) had VLS and 66.5% (95% CI: 59.8%–73.2%) had undetectable VL. Among 262 HIV-exposed children, 50.8% (95% CI: 42.8%–58.8%) received EID testing within 2 months of birth, whereas 17.9% (95% CI: 11.9%–23.8%) did not receive EID testing. Of 190 HIV-exposed children with a reported HIV test result, 2.1% (95% CI: 0.0%–4.6%) had positive results.

**Conclusions:**

MPHIA data demonstrate high PMTCT uptake at a population level. However, our results identify some gaps in VLS in postpartum women and EID testing.

## Introduction

In 2018, HIV prevalence in Malawi was estimated to be 9.2% among adults aged 15–49 years, with higher prevalence among women of reproductive age, similar to other epidemics in the region [1]. The fertility rate in Malawi is high, and with a 7% HIV prevalence reported in routine HIV testing in antenatal care (ANC) clinics in 2018, an estimated 45,000 HIV-positive mothers need antiretroviral drugs (ARVs) for prevention of mother-to-child transmission of HIV (PMTCT) annually [1–3].

Malawi has been at the forefront of implementing innovative PMTCT policies and services. In 2011, Malawi was the first country to transition the national PMTCT program to the Option B+ strategy, enrolling all pregnant and breastfeeding women on life-long antiretroviral therapy (ART), regardless of CD4 count or clinical stage [4, 5]. By the end of 2012, Option B+ was provided at 641 integrated PMTCT/ART sites, and the number of pregnant and breastfeeding women initiating ART increased by 748% [6]. As the program continued to mature, mother-to-child transmission of HIV (MTCT) decreased; in a national evaluation of the Option B+ program conducted between 2014 and 2016, early infant transmission was estimated to be 3.7% [7]. In addition, the 2015–2016 Malawi Population-based HIV Impact Assessment (MPHIA) reported high levels of awareness of HIV positive status and ART uptake during pregnancy among women who had a live birth in the 12 months before the survey [8].

Alongside PMTCT program scale-up, Malawi initiated and expanded early infant diagnosis (EID) testing for HIV-exposed infants born to HIV-positive women, with national guidelines recommending testing for HIV-exposed infants at the first opportunity by age 2 months, and ages 12 and 24 months [9]. In the last quarter of 2018, 9,834 specimens were tested with DNA-PCR for EID in the national laboratory system [2].

Despite the successes of Malawi’s PMTCT and EID programs, challenges remain in reaching Joint United Nations Programme on HIV/AIDS (UNAIDS) 90-90-90 targets (90% of HIV-positive individuals are aware of their HIV status; of these, 90% are receiving ART; and of these, 90% have viral load suppression [VLS]) among pregnant and breastfeeding women. MTCT rates in Malawi are still above those in other resource-constrained countries, which may reflect late ART initiation and issues with adherence [7]. In 2018, routine program data estimated that 71% of women initiating ART through Option B+ were retained at 12 months, similar to the 72% overall 12-month retention reported among adults in the national ART program [2]. In addition, the national PMTCT evaluation showed losses at each step of the PMTCT cascade, particularly in follow-up of HIV-exposed infants [7, 10]. The 2015–2016 MPHIA reported that less than half of infants born to HIV positive women were tested for HIV within 2 months of birth, and less than one-third were tested between 2 and 12 months of birth [8]. The successes associated with high coverage of PMTCT may be offset by inadequate adherence and retention on ART, undiagnosed incident HIV infections in late pregnancy or during breastfeeding, and low uptake of EID services; these factors may lower the rate of VLS and contribute to MTCT during the postpartum and breastfeeding periods [11].

While the 2015–2016 MPHIA reported high levels of HIV status awareness and ART among women reporting a live birth in the previous 12 months, we conducted this study to assess whether achievement of targets differed among sub-populations of women. This analysis utilized 2015–2016 MPHIA data to describe progress toward the UNAIDS 90-90-90 targets among the following groups of HIV-positive women: 1) had a live birth in the 3 years before the survey, 2) tested HIV-positive during the survey but reported being HIV-negative during their most recent pregnancy in the 3 years before the survey 3) were breastfeeding at the time of the survey, and 4) were pregnant at the time of the survey. Awareness of HIV positive status, ART uptake, and VLS, including undetectable viral load (VL), were assessed among women in these four groups to understand progress and gaps between each cascade step and identify areas in which achievement of 90-90-90 targets was being facilitated or hindered. For women who were aware of their HIV-positive status during their most recent pregnancy, data on their HIV-exposed infants’ HIV testing and prophylaxis were also analyzed to assess EID outcomes.

## Methods

The MPHIA was a nationally representative household survey conducted from November 2015 to August 2016 [8]. Households were sampled using a two-stage, stratified cluster sample design, and all adults aged 15–64 years residing in sampled households or visitors who slept in the household the night before the survey were eligible for participation. Eligible individuals were asked to participate in interviews and provide blood specimens for home-based and laboratory testing. In addition, biomarker procedures among children aged less than 15 years were conducted in half of the sampled households.

Consenting participants answered questions on sociodemographic characteristics, HIV testing, HIV care and treatment, and sexual behavior. Women were asked about their reproductive history and the most recent pregnancy resulting in a live birth in the 3 years before the survey. Women who reported a live birth in the 3 years before the survey answered questions on ANC, awareness of HIV status (HIV testing before pregnancy, during pregnancy, or did not receive an HIV test), ART use among HIV-positive women during pregnancy (initiated ART before pregnancy, initiated ART during pregnancy, or did not receive ART) and postpartum, breastfeeding status at the time of the survey, and infant HIV testing. Respondents were also asked about sexual partnerships, HIV status of partners, and disclosure of status between the respondent and their partner. For women who identified sexual partners in the household who participated in the survey, partner disclosure of HIV status to the woman was assessed using the partner’s interview data and HIV status of the partner was based on the MPHIA test result.

Following the interview, those who consented for blood collection (venous blood draw) and HIV testing received home-based HIV testing and counseling using the national sequential rapid testing algorithm, composed of Determine HIV-1/2 (Abbott Molecular Inc., Des Plaines, IL United States) as the screening test and Uni-Gold (Trinity Biotech, Wicklow, Ireland) testing for all reactive specimens. All specimens testing positive with both tests at the household were confirmed with Geenius HIV 1/2 Supplemental Assay (Bio-Rad, Hercules, CA United States) at a satellite laboratory.

HIV-1 VL measurement was performed on plasma specimens for all confirmed HIV-positive participants using the Abbott RealTime HIV-1 assay on the m2000 System. For participants with insufficient volumes of plasma samples or for whom venous blood specimens could not be collected, VL was measured using dried blood spot (DBS) samples using the open-mode protocol for the Abbott Real Time HIV-1 assay. Additionally, testing of infants aged <18 months was conducted using Determine as a screening test and then DNA-PCR testing if the Determine result was positive. DNA-PCR testing was performed on DBS samples from HIV-exposed children using the Abbott Real Time HIV-1 qualitative assay. VL and DNA-PCR results were returned to health facilities selected by HIV-positive participants and children’s parents or guardians, respectively.

For participants with confirmed HIV-positive status, DBS specimens were tested for presence of selected ARVs using high-resolution liquid chromatography coupled with tandem mass spectrometry [12]. Three ARVs were selected as markers for the most prescribed first-line and second-line regimens in Malawi: efavirenz, atazanavir, and lopinavir. Samples from participants who had VLS or who self-reported receiving ART, but who had no evidence of these three compounds, were tested for nevirapine.

Analyses were restricted to women aged 15–64 years reporting a live birth in the 3 years before the survey or women who were pregnant at the time of the survey who consented for blood collection and HIV testing and tested HIV-positive during the survey. We calculated progress toward the UNAIDS 90-90-90 targets among the following groups of women who were confirmed as HIV positive during MPHIA: 1) those who had a live birth in the 3 years before the survey, 2) those who reported being HIV-negative during the most recent pregnancy in the 3 years before the survey, 3) those who reported breastfeeding at the time of the survey, and 4) those who were pregnant at the time of the survey. Awareness of HIV-positive status was based on self-report or presence of ARVs in blood. ART use was based on self-report or presence of detectable ARVs in blood among those who were aware of their HIV-positive status. We analyzed overall VL among women who delivered in the previous 3 years and who tested HIV-positive in the survey using both VLS defined as <1,000 copies/mL and undetectable VL defined as target not detected or below the level of detection by the Abbott system (<40 copies/mL). We described the last-born children of women who reported being HIV positive during the most recent pregnancy in the 3 years before the survey.

Survey data analyses were conducted in Stata version 16 (StataCorp, College Station, TX USA) and in R version 4.0 using the *survey* package [13, 14]. Weighted estimates were calculated accounting for the 2-stage cluster sample design and non-response using a Chi-square Automatic Interaction Detection (CHAID) tree classification scheme. Jackknife replication weights were used for variance estimation and calculating 95% confidence intervals (CI). For the EID analysis, unadjusted prevalence ratios, 95% confidence intervals (CI) and p-values were estimated using the survey-weighted generalized linear model (svyglm) function in the survey package in R. P-values <0.05 were considered statistically significant.

Ethical review and approvals for all consent and study procedures were obtained from the National Health Sciences Research Committee in Malawi and the US Centers for Disease Control and Prevention and Columbia University Institutional Review Boards. All participants aged 18–64 years provided written consent. Parental or guardian written assent was requested for participation of children aged 0–17 years, and written assent was requested from children who were aged 10–17 years.

## Results

**Fig 1:**
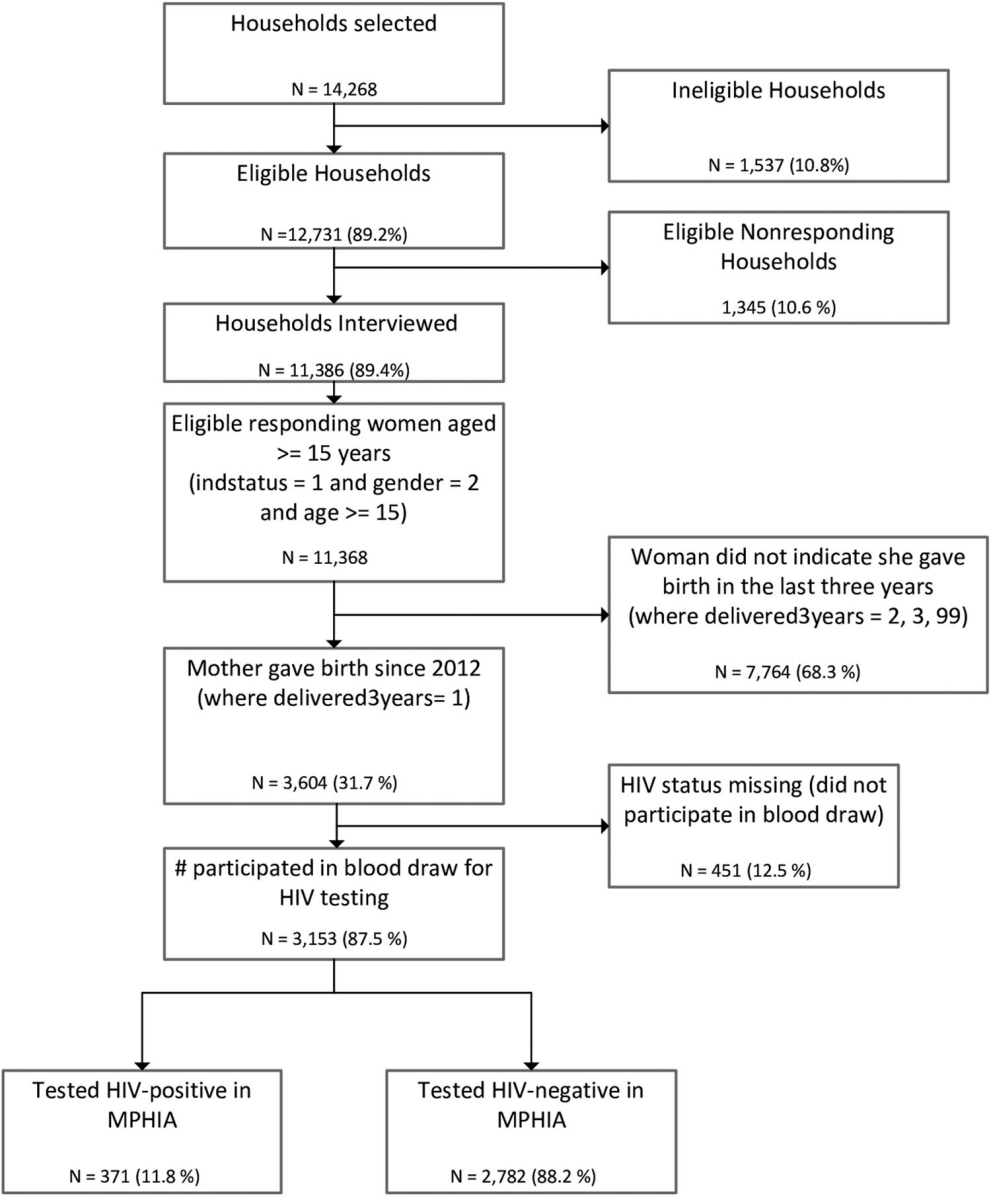
Participant flowchart, 2015–2016 Malawi Population-based HIV Impact Assessment (MPHIA) survey.

Of the 3,604 eligible women who reported giving birth within the 3 years before the survey, 3,153 (87.5%) participated in HIV testing, and 371 (10.1%, 95% CI: 8.8%–11.3%) tested HIV positive (Fig 1). Of these 371 HIV-positive women, median age was 30 years (interquartile range, 26–35 years; range, 17–52 years), 65.8% (95% CI: 59.1%–72.4%) had completed primary education, and nearly all (98.8%, 95% CI: 97.4%–100.0%) reported receiving ANC during their most recent pregnancy (Table 1). Of the 367 women who reported receiving ANC and being tested for HIV during their most recent pregnancy, less than half (44.9%, 95% CI: 39.3%–50.5%) reported that they knew their HIV-positive status before the pregnancy, 27.5% (95% CI: 21.4%–33.6%) reported testing HIV positive during the pregnancy, 6.7% (95% CI: 3.9%–9.5%) were not aware of their HIV status during pregnancy, and 19.7% (95% CI: 14.7%–24.6%) reported testing HIV negative during the pregnancy. Of the 278 women who reported being HIV positive during their most recent pregnancy, 52.2% (95% CI: 45.3%–59.1%) were receiving ART at their first ANC visit and 46.3% (95% CI: 39.4%–53.2%) initiated ART during pregnancy or labor. Among the 271 women who reported ART use during pregnancy, 88.6% (95% CI: 83.6%–93.5%) had VLS (Table 1).

**Table 1:** Characteristics of HIV-positive women aged 15–64 years who had a live birth in the 3 years before the 2015–2016 Malawi Population-based HIV Impact Assessment (MPHIA) survey (N = 371).

Characteristics	Weighted percentage (95% CI)	N^1^
**Age (years), median (interquartile range)**	30.0 (26.0–35.0)	371
**Age group, years**		
15–24	16.2 (11.8–20.6)	67
25–34	58.2 (52.0–64.3)	208
≥35	25.6 (20.4–30.8)	96
**Education status**		
No education	12.5 (8.1–16.8)	39
Primary	65.8 (59.1–72.4)	233
Secondary	20.3 (15.2–25.4)	91
More than secondary	1.4 (0.3–2.6)	8
**Live births in the 3 years before the survey**		
1	78.4 (73.4–83.5)	297
≥1	21.6 (16.5–26.6)	74
**Antenatal care during most recent pregnancy**		
Received antenatal care	98.8 (97.4–100.0)	367
Did not receive antenatal care	1.2 (0.0–2.6)	4
**HIV testing during most recent pregnancy (N = 367)**		
Already knew HIV-positive status before pregnancy	44.9 (39.3–50.5)	169
Tested HIV-positive during pregnancy	27.5 (21.4–33.6)	109
Tested HIV-negative during pregnancy	19.7 (14.7–24.6)	68
Not aware of HIV status^2^	6.7 (3.9–9.5)	21
**ART use during most recent pregnancy (N = 278)** ^**3**^		
On ART at first antenatal visit	52.2 (45.3–59.1)	139
Newly initiated ART during pregnancy or labor	46.3 (39.4–53.2)	132
Not on ART	1.5 (0.1–2.9)	7
**Viral suppression (N = 271)** ^**4**^		
Viral load <1000 copies/mL	88.6 (83.6–93.5)	238
Viral load ≥1000 copies/mL	11.4 (6.5–16.4)	33
**Mother disclosed HIV status to partner (N = 278)** ^**3**^		
Yes	60.1 (53.5–67.9)	181
No	39.3 (32.1–46.5)	97
**Partner HIV status (N = 210)** ^**5**^		
Positive	38.2 (30.3–46.1)	75
Negative	15.4 (9.2–21.8)	33
Unknown	46.3 (38.2–54.4)	102
**Partner disclosed HIV-positive status to mother (N = 75)** ^**6**^		
Yes	66.6 (52.9–80.3)	45
No	33.4 (19.7–47.1)	30

^1^ May not add up to total due to missing values. Variables with restricted denominators are explained below.

^2^ Tested for HIV during pregnancy but did not receive result, or did not test for HIV during pregnancy and did not know they were HIV positive before pregnancy.

^3^ Among women who knew they were HIV positive before most recent pregnancy or tested HIV positive during most recent pregnancy.

^4^ Among women who were receiving ART before their first antenatal visit or initiated ART during most recent pregnancy.

^5^ Among women who reported a sexual partner in the past 12 months. HIV status of the partner is based on HIV testing for MPHIA. The unknown category comprises cohabiting sexual partners who did not participate in MPHIA HIV testing.

^6^ Among women whose partner tested HIV positive in MPHIA. Partner’s interview data were analyzed to determine if the partners disclosed their HIV status to the women.

Abbreviations: CI, confidence interval; ART, antiretroviral therapy.

### 90-90-90 cascade in HIV-positive women who delivered within the 3 years before the survey

Among the 371 women who delivered within 3 years and tested HIV-positive during the survey, 322 (84.2%, 95% CI: 79.7%–88.6%) were aware of their status at the time of the survey, 308 (79.9%, 95% CI: 74.8%–85.0%) were receiving ART, and 273 (72.9%, 95% CI: 67.4%–78.4%) had VLS. Of the 322 women who reported knowing their HIV positive status, 94.9% (95% CI: 91.7%–98.2%) were receiving ART, and of these, 91.2% (95% CI: 87.6%–94.8%) had VLS (Fig 2.1).

**Fig 2:**
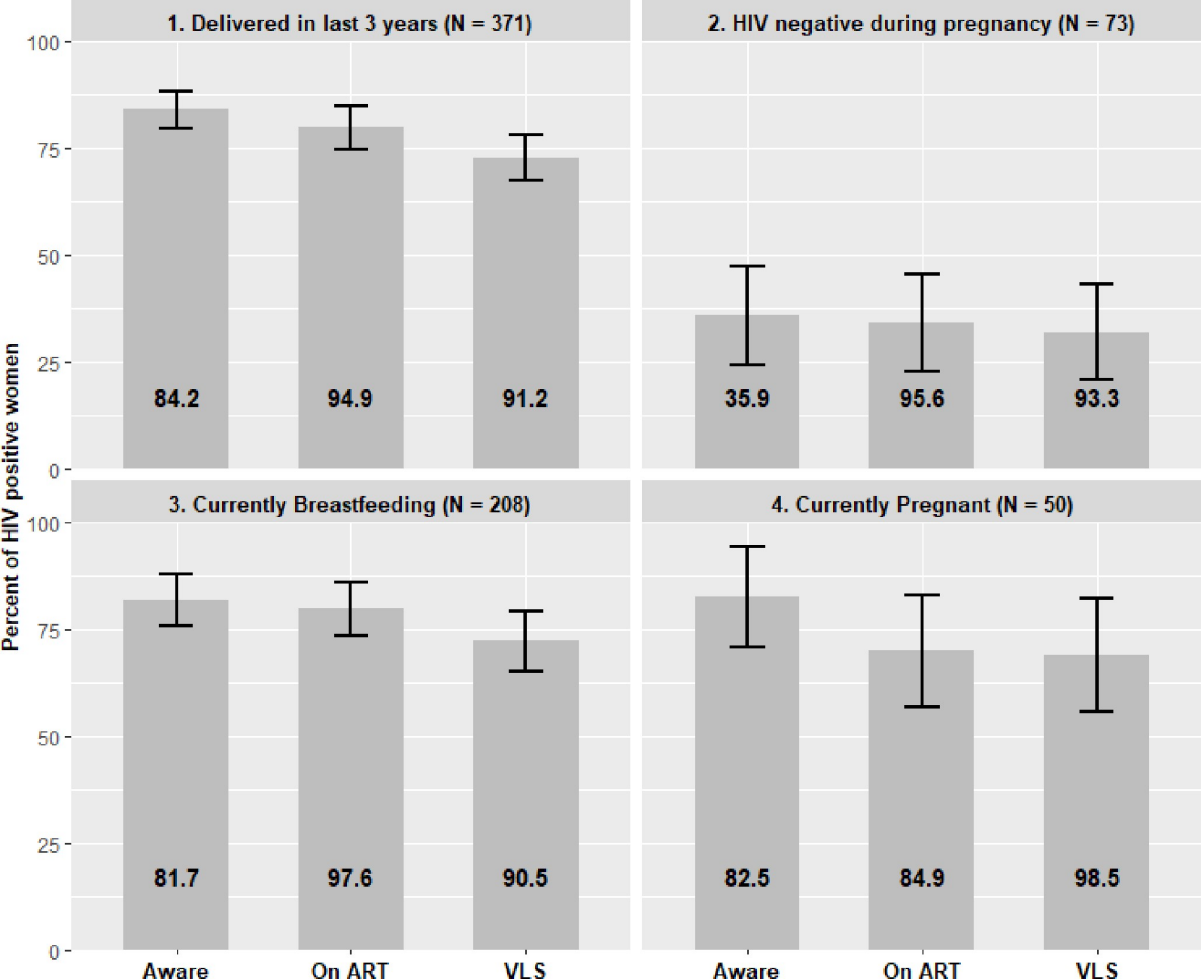
Joint United Nations Programme on HIV/AIDS 90-90-90 cascades among HIV-positive women aged 15–-64 years, Malawi Population-based HIV Impact Assessment (MPHIA) survey (2015–-2016). 1) Women who had a live birth in the 3 years before the survey. 2) Women who reported being HIV negative during the last pregnancy. 3) Women who were breastfeeding at the time of the survey. 4) Women who were pregnant at the time of the survey.

Of the 371 women who tested HIV positive during the survey, 73 (19.7%) reported an HIV-negative status during the most recent pregnancy in the 3 years before the survey. Of these 73 women, 30 (35.9%, 95% CI: 24.4%–47.4%) were aware of their HIV-positive status at the time of the survey, 28 (34.3%, 95% CI: 22.9%–45.7%) were receiving ART, and 24 (32.0%, 95% CI: 20.8%–43.2%) had VLS. Of the 30 women who were aware of their HIV-positive status, 95.6% (95% CI: 89.3%–100.0%) were receiving ART, and of these, 93.3% (95% CI: 85.5%–100.0%) had VLS (Fig 2.2).

Among 371 women who delivered in the previous 3 years and who tested HIV-positive during the survey, 283 (76.0%, 95% CI: 70.4%–81.7%) had VLS, and 256 (66.5%, 95% CI: 59.8%–73.2%) had undetectable VL (Table 2). VLS or undetectable VL varied significantly by age, HIV status during pregnancy, disclosure of partner’s HIV status, and current ART use. In women whose partners tested HIV positive in the survey, 87.6% (95% CI: 80.2%–95.0%) had VLS and 73.9% (95% CI: 61.5%–86.2%) had undetectable VL when the partner reported that they had disclosed their HIV-positive status to the woman. Among women who were currently breastfeeding, 74.4% (95% CI: 67.0%–82.0%) and 66.0% (95% CI: 57.7%–74.3%) had VLS and undetectable VL, respectively. VLS and unsuppressed VL were observed in 75.2% (95% CI: 67.9%–82.5%) and 64.2% (95% CI: 55.7%–72.8%) of women who had delivered more than 12 months before the survey, compared to 77.8% (95% CI: 69.9%–85.6%) and 70.8% (95% CI: 61.5%–80.1%) who had delivered 12 months or less before the survey, respectively.

**Table 2:** Viral load suppression among HIV-positive women aged 15–64 years who had a live birth in the 3 years before the 2015–2016 Malawi Population-based HIV Impact Assessment (MPHIA) survey (N = 371).

		Virally suppressed (viral load<1000 copies/mL)				Undetectable viral load (target not detected)			
	N	Unweighted n	Weighted %	95% CI	p-value	Unweighted n	Weighted %	95% CI	p-value
**All**	371	283	76.0	70.4–81.7		256	66.5	59.8–73.2	
**Age, years**					<0.001				0.01
15–24	67	45	64.1	47.6–80.6		43	61.4	44.9–78.0	
25–34	208	153	72.5	64.8–80.1		135	61.0	51.9–70.0	
35–64	96	85	91.7	85.5–97.8		78	82.2	73.3–91.1	
**Residence**					0.3				0.5
Urban	164	117	71.5	62.2–80.7		109	63.4	54.3–72.5	
Rural	207	166	77.4	70.6–84.1		147	67.4	59.1–75.6	
**Education status**					0.6				0.07
No education	39	32	80.4	64.8–96.1		32	80.4	64.8–96.1	
Primary	233	182	77.1	70.4–83.7		164	67.0	59.1–74.9	
Secondary	91	62	69.5	57.2–81.7		53	55.0	41.5–68.4	
More than secondary	8	7	83.5	46.8–100.0		7	83.5	46.8–100.0	
**HIV testing during most recent pregnancy**					<0.001				<0.001
Already knew HIV-positive status before pregnancy	169	150	91.1	86.2–96.1		141	84.0	77.0–90.9	
Tested HIV-positive during Pregnancy	109	90	82.4	72.2–92.6		80	68.2	56.4–80.0	
Tested HIV-negative during Pregnancy	68	30	47.4	33.6–61.3		23	36.4	21.5–51.3	
Not aware of HIV status^1^	21	10	33.2	12.3–54.1		9	28.9	8.9–48.9	
**ART use during most recent pregnancy** ^**2**^					0.3				0.06
On ART at first antenatal visit	139	123	90.9	85.5–96.4		116	84.1	76.9–91.4	
Newly initiated ART during pregnancy or labor	132	115	85.9	77.3–94.6		104	72.6	62.2–83.1	
**Mother disclosed HIV-positive status to partner** ^**2**^					0.8				0.5
Yes	181	156	88.4	82.6–94.1		145	79.6	71.1–87.4	
No	97	84	87.0	77.8–96.0		76	75.6	64.5–86.7	
**Partner HIV status** ^**3**^					0.5				0.3
Positive	75	59	75.2	62.9–87.5		52	63.5	50.4–76.7	
Negative	33	21	65.6	44.5–86.7		18	52.7	30.0–75.4	
Unknown	102	78	78.5	68.8–88.2		71	69.4	58.5–80.4	
**Partner disclosed HIV-positive status to mother** ^**4**^					<0.001				0.02
Yes	45	40	87.6	80.2–95.0		35	73.9	61.5–86.2	
No	30	19	50.4	24.4–76.5		17	43.0	18.3–67.6	
**Current ART use** ^**5**^					<0.001				<0.001
Yes	308	273	91.2	87.4–95.0		252	81.6	76.1–87.1	
No	63	10	15.8	6.1–25.5		4	6.4	0.0–13.4	
**Breastfeeding status** ^**6**^					0.7				0.4
Currently breastfeeding	208	157	74.4	67.0–82.0		143	66.0	57.7–74.3	
Ever breastfed but not Currently	143	109	76.9	67.5–86.4		100	67.9	57.6–78.2	
**Time since most recent delivery**					0.6				0.3
≤12 months	130	101	77.8	69.9–85.6		94	70.8	61.5–80.1	
>12 months	241	182	75.2	67.9–82.5		162	64.2	55.7–72.8	

^1^ Tested for HIV during pregnancy but did not receive result, or did not test for HIV during pregnancy and did not know they were HIV positive before pregnancy.

^2^ Among women who knew they were HIV positive before most recent pregnancy or tested HIV positive during pregnancy; excluded the category “not on ART” (N = 5).

^3^ Among women who reported a sexual partner in the past 12 months. HIV status of the partner is based on HIV testing for MPHIA. The unknown category comprises cohabiting sexual partners who did not participate in MPHIA HIV testing.

^4^ Among women whose partner tested HIV-positive in MPHIA (N = 75). Partner’s interview data were analyzed to determine if the partner disclosed their HIV status to the women.

^5^ Self-reported in the survey or antiretroviral drugs detected.

^6^ Excluded women who reported that they never breastfed (N = 5) and women whose breastfeeding status was unknown (N = 15).

Abbreviations: CI, confidence interval; ART, antiretroviral therapy.

### 90-90-90 cascade among women currently breastfeeding at the time of the survey

Among the 208 HIV-positive women who were currently breastfeeding at the time of the survey, 179 (81.7%, 95% CI: 75.6%–87.9%) were aware of their HIV-positive status, 174 (79.8%, 95% CI: 73.5%–86.1%) were receiving ART, and 153 (72.2%, 95% CI: 65.1%–79.3%) had VLS. Of the 179 women who were aware of their HIV-positive status, 97.6% (95% CI: 94.9%–100.0%) were receiving ART; of the 174 women receiving ART, 90.5% (95% CI: 85.8%–95.1%) had VLS (Fig 2.3).

### 90-90-90 cascade among women pregnant at the time of the survey

Among 50 HIV-positive women who were pregnant at the time of the survey, 42 (82.5%, 95% CI: 70.6%–94.3%) were aware of their HIV-positive status, 37 (70.0%, 95% CI: 56.9%–83.1%) were receiving ART, and 36 (68.9%, 95% CI: 55.7%–82.1%) had VLS. Of the 42 women who were aware of their HIV-positive status, 84.9% (95% CI: 72.2%–97.6%) were receiving ART, and of the women receiving ART, 98.5% (95% CI: 95.4%–100.0%) had VLS (Fig 2.4).

### HIV-exposed infant characteristics and EID testing

Among 263 HIV-exposed children born to women who reported being HIV positive during their most recent pregnancy in the 3 years before the survey, mothers reported that 95.0% (95% CI: 91.7%–98.3%) were delivered at a health facility, 69.4% (95% CI: 62.4%–76.4%) received nevirapine prophylaxis, 82.2% (95% CI: 76.4%–88.1%) received cotrimoxazole, and 65.9% (95% CI: 58.9%–72.9%) were being breastfed at the time of the survey (Table 3).

**Table 3:** Characteristics of HIV-exposed children^1^ aged ≤36 months included in the 2015–2016 Malawi Population-based HIV Impact Assessment (MPHIA) survey (N = 263).

Characteristics	Weighted percentage (95% CI)	N^2^
Child age (months), median (IQR)	17.0 (6.0–25.0)	263
Child age, months		
≤6	25.1 (19.3–31.0)	63
7–12	13.3 (8.9–17.6)	37
13–24	35.8 (29.0–42.7)	98
24–36	25.7 (19.4–32.1)	65
Place of delivery (N = 253)		
Home	3.1 (0.4–5.8)	7
Health Facility	95.0 (91.7–98.3)	241
Other	2.0 (0.0–3.8)	5
Received nevirapine prophylaxis (N = 252)		
Yes	69.4 (62.4–76.4)	177
No	30.1 (23.1–37.2)	75
Received cotrimaxazole (N = 251)		
Yes	82.2 (76.4–88.1)	207
No	16.4 (10.9–21.9)	44
Breastfeeding status (N = 262)		
Never breastfed	0.8 (0.0–2.0)	3
Ever breastfed but not currently breastfeeding	32.8 (25.7–39.8)	86
Currently breastfeeding	65.9 (58.9–72.9)	173
Breastfeeding child age (months, N = 173)^3^		
≤6	100.0 (100.0–100.0)	63
7–12	98.6 (95.8–100.0)	36
13–24	67.1 (56.6–77.5)	62
24–36	14.0 (4.6–23.4)	12
Timing of last virologic test of child (N = 262)		
Within 2 months of birth	50.8 (42.8–58.8)	145
>2 months and <12 months of birth	28.0 (21.3–34.7)	69
>12 months of birth	1.2 (0.0–2.8)	3
HIV test done, but the date of test unknown	2.1 (0.1–4.1)	5
Did not undergo HIV testing	17.9 (11.9–23.8)	40
Virologic test result (N = 263)		
Positive	1.4 (0.0–3.2)	3
Negative	67.0 (59.7–74.2)	187
Don’t know	13.5 (7.8–18.9)	32
Missing	18.2 (12.3–24.2)	41
Virologic test result of lastborn child (N = 190)^4^		
Positive	2.1 (0.0–4.6)	3
Negative	97.9 (95.4–100.0)	187
Child tested for HIV after mother stopped breastfeeding (N = 85)^5^		
Yes	67.4 (55.4–79.5)	56
No	32.6 (20.5–44.6)	29
Maternal ART use during pregnancy (N = 258)		
Mother already on ART at first antenatal visit	53.3 (46.6–60.0)	130
Mother newly initiated on ART during pregnancy or Labor	45.2 (38.5–51.9)	123
Mother not on ART during pregnancy or labor	1.5 (0.0–3.1)	5

^1^ Mother reported being HIV-positive during pregnancy; child is the lastborn child from the most recent pregnancy in the 3 years before the survey.

^2^ May not add up to total due to missing values. Variables with restricted denominators are explained below.

^3^ Among children whose mothers reported currently breastfeeding.

^4^ Virologic test result of the lastborn child restricted to women who provided a positive or negative response.

^5^ Among women who breastfed their child but were not breastfeeding at the time of the survey.

Abbreviations: CI, confidence interval; IQR, interquartile range; EID, early infant diagnosis; ART, antiretroviral therapy.

Complete data on receipt and timing of EID testing were available for 262 of the 263 HIV-exposed children. Of these 262 children, 50.8% (95% CI: 42.8%–58.8%) were reported to have received an EID test within 2 months of birth, 28.0% (95% CI: 21.3%–34.7%) were tested 2–12 months after birth, 1.2% (95% CI: 0.0%–2.8%) were tested 12 months after birth, and 17.9% (95% CI: 11.9%–23.8%) did not undergo EID testing. Of 190 HIV-exposed children with a reported HIV test result, 2.1% (95% CI: 0.0%–4.6%) had a positive virologic test. HIV testing after the mother stopped breastfeeding was done in 67.4% (95% CI: 55.4%–79.5%) of the children (Table 3).

Infants receiving an EID test by 2 months of age differed by urban versus rural residence: infants of women residing in rural areas were less likely to receive an EID test by 2 months than infants of women in urban areas (p = 0.004). Infant EID testing differed by educational status of the mother, with infants of mothers with secondary or higher education more likely to be tested by age 2 months. EID testing of their HIV-exposed infants by age 2 months was more commonly reported by younger women (aged 15–24 years), among women already receiving ART at their first ANC visit, and among those who disclosed their HIV-positive status to a spouse or partner; however, the analysis did not find that EID testing differed between these groups (Table 4).

**Table 4:** Factors associated with receiving early infant diagnosis (EID) testing within 2 months of birth^1^ among HIV-exposed children^2^ included in the 2015–2016 Malawi Population-based HIV Impact Assessment (MPHIA) survey (N = 262).

Characteristic	N^3^	Received EID within 2 months of birth, weighted percentage	95% CI	Unadjusted prevalence ratio	95% CI	p-value
Overall	262	50.8	42.8–58.8			
Mother’s age, years						
15–24	47	64.1	45.3–82.9	1	-	-
25–34	139	48.5	38.3–58.8	0.8	0.5–1.1	0.11
≥35	76	47.1	33.6–60.6	0.7	0.5–1.0	0.13
Residence						
Urban	123	66.4	56.3–76.5	1		
Rural	139	45.7	35.8–55.5	0.7	0.5–0.9	0.004
Mother’s education						
No education	30	41.1	20.1–62.1	1		
Primary	169	47.9	38.1–57.7	1.2	0.7–2.0	0.6
Secondary or more	63	68.3	54.0–82.5	1.7	1.0–2.8	0.06
Mother’s timing of ART initiation^4^						
Already on ART at first antenatal visit	130	53.8	42.7–64.9	1		
Newly initiated on ART during pregnancy or labor	124	48.2	37.2–59.2	0.9	0.7–1.2	0.45
Mother disclosed HIV status to spouse/partner						
Yes	174	52.4	42.8–61.5	1.1	0.8–1.5	0.60
No	88	48.2	42.8–62.0	1		
Partner HIV status^5^						
Positive	60	56.5	40.7–72.4	1		
Negative	19	49.7	18.9–80.4	0.9	0.5–1.7	0.70
Unknown	80	61.0	47.4–74.7	1.1	0.8–1.5	0.65

^1^ Mother reported EID testing of HIV-exposed infant.

^2^ Mother reported being HIV-positive during pregnancy; child is the lastborn child from the most recent pregnancy in the 3 years before the survey.

^3^ Restricted to non-missing responses to the timing of lastborn child’s last virologic test.

^4^ Among women who reported ART use during pregnancy.

^5^ Among women who reported a sexual partner in the past 12 months. HIV status of the partner is based on HIV testing for MPHIA. The unknown category comprises cohabiting sexual partners who did not participate in MPHIA HIV testing.

Abbreviations: CI, confidence interval; ART, antiretroviral therapy.

## Discussion

The findings of the 2015–2016 MPHIA survey demonstrate the success of Malawi’s PMTCT program. In this representative sample of Malawian women with a live birth in the 3 years before the survey and among those who were currently pregnant, coverage and uptake of ANC and HIV testing were high. Though awareness of HIV-positive status did not reach the 90% target, awareness was higher among pregnant and postpartum women than in the general population [8]. Of those who reported awareness of their HIV-positive status, most reported receiving ART and had VLS, indicating high rates of ART adherence at the population level. However, our analysis identified a few challenges: knowledge of HIV-positive status among pregnant and breastfeeding women and timely EID remained the largest gaps. In addition, low VLS rates among some subgroups of pregnant and postpartum women indicate poor ART retention and adherence and/or suboptimal drug regimens.

Since Malawi’s national PMTCT program was established in 2003, the program has experienced rapid growth and scale-up. The adoption of Option B+ in 2011 was a major global breakthrough in PMTCT service delivery and was a forerunner of the World Health Organization (WHO)-recommended *Test and Treat* strategy. However, at the time Option B+ was being considered, concerns were raised around retention of women initiating ART through Option B+ [5]. These nationally representative household-based survey results for VLS prevalence among women who knew their HIV-positive status and were receiving ART indicate good retention, consistent with facility-based studies in Malawi [15–17], with higher VLS among those who had initiated ART before pregnancy. The high VLS rates among breastfeeding women also indicates good retention and adherence during this critical period, though awareness of HIV status in this group did not reach the UNAIDS 90% target. Overall, ART uptake among those aware of their HIV-positive status and VLS among those on ART exceeded the UNAIDS 90% targets, demonstrating that once women receive an HIV diagnosis, they access treatment and have VLS.

Conversely, VLS rates were lowest among women who reported being HIV-negative during their most recent pregnancy in the 3 years before the survey but who tested HIV-positive during the survey. Although this sub-group was small and time of infection cannot be established, with 97% of women giving birth in the 12 months before the survey reporting knowing their HIV status, it may be that HIV-positive women who reported being HIV-negative represent incident infections [8]. In addition, other studies have found higher HIV incidence among pregnant and postpartum women, likely due to both biological and behavioral factors [18–20]. These results indicate that increasing accessibility of HIV prevention and testing services in the postpartum period will be important to achieve WHO pediatric HIV elimination targets [18, 21]. Such approaches could address incident cases occurring during late pregnancy and breastfeeding; though small in number, the women with new HIV diagnoses during MPHIA may represent a considerable proportion of the infant infections in the population overall [22–24].

While two-thirds of our participants had undetectable VL, some women in the VLS group had detectable viremia, possibly indicating imperfect adherence and/or development of drug resistance and treatment failure. Analyses of low-detectable VL (40–1,000 copies/mL) and undetectable VL among postpartum women in Malawi and South Africa found associations between sub-optimal adherence and low-detectable VL, and demonstrated increased risk of MTCT among women with low-detectable VL compared to those with undetectable VL [25, 26]. Continued efforts to improve adherence, including routine VL monitoring, may ensure that women have undetectable VL. The 2019 transition to dolutegravir-based ART regimens with a higher barrier to resistance development could improve long-term VLS among women in Malawi’s PMTCT program.

We also found EID prophylaxis and testing among HIV-exposed infants to be a significant gap in the PMTCT cascade. Although the PMTCT program has made significant progress toward testing and managing HIV-positive pregnant women, the incorporation and coverage of EID services has been hindered by loss to follow-up, limited laboratory capacity, and reliance on centralized PCR laboratories [27–29]. Despite established EID guidelines and procedures, challenges have continued after the survey [27, 30, 31]. In 2019, Malawi’s national VL testing recommendations shifted from testing once every 2 years to annual testing for patients receiving ART, further burdening the laboratory system [32]. Given the limited capacity of Malawi’s laboratory infrastructure, incorporating point-of-care technology for DNA-PCR may significantly improve timely uptake of EID.

Finally, our analysis found that disclosure of the mother’s HIV-positive status to her partner may be associated with undetectable VL and timely EID testing of their infants, suggesting that facilitating disclosure may improve outcomes for both mothers and their children. Offering HIV-positive mothers assistance with disclosure to their partners and other community and family support systems to assist with ART adherence and follow-up of their HIV-exposed children could help improve outcomes.

This analysis is subject to several limitations. MPHIA was a cross-sectional household survey; thus, causality and timing of infection cannot be established based on these data. Women were asked about their most recent pregnancy resulting in a live birth in the 3 years before the survey, which may have resulted in recall bias. In addition, this approach would exclude previous live births among women who had multiple live births in this period. Outcomes and cofactors reported on the children in this analysis only pertain to children who were alive at the time of the survey; deceased children were excluded from this analysis, which may bias these results. Also, because Determine was used as a screening test for infants aged <18 months before proceeding with PCR testing if the Determine result was positive, using this screening approach may have missed some HIV-positive infants who tested negative using Determine. In addition, during the 3 years before the survey, HIV prevention and treatment programs evolved rapidly, and these results reflect program performance during 2013–2015. Changes in programmatic coverage during this period could not be fully reflected in the analysis. Finally, data on HIV status awareness and ART uptake were self-reported, which may have led to under-reporting of these indicators due to stigma. However, the use of biomarkers, including VL measurements and ART testing, should minimize misclassification and generally confirmed self-reported data.

MPHIA’s nationally representative results indicate that Malawi’s PMTCT program accomplishments among women of reproductive age are a significant contribution to Malawi’s progress toward achieving the UNAIDS 90-90-90 targets. Continued efforts are needed to identify HIV-positive pregnant and breastfeeding women, including incident cases in late pregnancy and postpartum periods, to ensure that these women are receiving ART and have VLS. In addition, innovations in EID programs may be considered to bring the uptake of EID services to levels similar to those of the national PMTCT program.
